# Sodium bicarbonate combined with urokinase for the prevention and management of hemodialysis central venous catheter dysfunction

**DOI:** 10.3389/fphar.2026.1775941

**Published:** 2026-07-06

**Authors:** Lin Lin, Lin-Jie Hu, Jia-Fei Geng, Ying Zhang, Zhi-Hui Li, Ye-Huan Xu

**Affiliations:** 1 Department of Blood Purification, The People’s Hospital of Huantai, Zibo City, Shandong, China; 2 Department of Neurology Ward 2, Huantai County People’s Hospital, Zibo City, Shandong, China

**Keywords:** catheter dysfunction, catheter lock solution, central venous catheter, hemodialysis, sodium bicarbonate, urokinase

## Abstract

**Background:**

Central venous catheter (CVC) dysfunction remains a common problem in maintenance hemodialysis and may impair prescribed blood flow and dialysis adequacy. This study aimed to evaluate whether a sodium bicarbonate–urokinase lock within a standardized dwell-and-aspiration protocol was associated with improved catheter patency and safety outcomes compared with a saline–urokinase lock.

**Methods:**

This retrospective study included 220 clinically stable adults undergoing maintenance hemodialysis with an indwelling CVC between December 2023 and March 2024. Patients were grouped according to the predefined catheter-locking schedule used in routine practice: sodium bicarbonate plus urokinase group (n = 115) and 0.9% sodium chloride plus urokinase group (n = 105). Urokinase was prepared at 10,000 U/mL, instilled at 1.1–1.2 times the catheter lumen volume, dwelled for 30 min, and followed by standardized negative-pressure aspiration. Outcomes included catheter dysfunction, event rates, dialysis performance, procedural response, recurrence, catheter exchange, and safety events. Multivariable logistic regression was used to adjust for clinically relevant covariates.

**Results:**

Catheter dysfunction occurred in 20.9% of patients in the sodium bicarbonate–urokinase group and 34.3% in the saline–urokinase group (P = 0.026). Dysfunction event rates were 4.35 versus 8.30 per 1,000 catheter-days, respectively (incidence rate ratio = 0.52, P = 0.002). After covariate adjustment, the sodium bicarbonate–urokinase regimen was associated with lower odds of dysfunction (adjusted odds ratio = 0.49, 95% confidence interval: 0.26–0.90; P = 0.024). Single-procedure patency restoration was more frequent (85.7% vs. 65.6%; P = 0.033), while repeat intervention and catheter exchange/reinsertion were less frequent. Bleeding events and laboratory safety indicators were comparable. Catheter-related bloodstream infection was infrequent but observed less often in the sodium bicarbonate–urokinase group (0.9% vs. 6.7%; P = 0.029).

**Conclusion:**

Sodium bicarbonate combined with urokinase within a standardized dwell-and-aspiration protocol may be associated with lower observed rates of CVC dysfunction, fewer dysfunction-related events, a higher observed rate of single-procedure patency restoration, and a comparable safety profile. Infection-related findings should be interpreted cautiously because of the small number of events and should be considered hypothesis-generating.

## 1 Introduction

Central venous catheters (CVCs) continue to be used in hemodialysis practice, particularly at dialysis initiation and when arteriovenous access is not available or cannot be used (Sohail et al., 2021; Wasse et al., 2008). Contemporary reviews highlight that catheter dependence remains frequent and is accompanied by clinically relevant complications that can compromise dialysis delivery and increase healthcare utilization (Chen et al., 2025). Catheter dysfunction, commonly operationalized by persistent inability to achieve prescribed extracorporeal blood flow, recurrent lumen occlusion, or pressure-alarm–driven interruption of treatment, represents a major noninfectious complication, with review-level estimates indicating that a substantial proportion of catheters may ultimately be removed due to dysfunction (Lazarus et al., 2025). The pathophysiology of catheter dysfunction is multifactorial and typically reflects intraluminal thrombosis, mural thrombus, and/or fibrin sheath formation, often interacting with catheter position, central venous stenosis, and flow-related factors (Xu et al., 2023). Catheter-related thrombosis is clinically important not only because it can precipitate access failure and procedural escalation, but also because thrombotic and inflammatory pathways may co-exist with infectious processes and biofilm, thereby linking dysfunction risk with downstream catheter-related adverse outcomes (López-Rubio et al., 2024). Imaging- and intervention-based evaluations of dysfunctional tunneled catheters further support that structural abnormalities and sheath-related phenomena are common contributors and frequently require targeted salvage strategies beyond routine troubleshooting (Jin et al., 2023; Wang et al., 2022).

Prevention and management strategies for catheter dysfunction include standardized catheter care, optimization of catheter tip position and blood flow dynamics, and selection of interdialytic lock solutions (Boucley et al., 2023). Heparin-based locks are widely used; however, uncertainty persists regarding the optimal concentration and the balance between maintaining patency and minimizing bleeding-related concerns, as reflected in recent quantitative syntheses comparing different heparin-locking concentrations (Mai et al., 2019). For non-tunneled catheters, systematic comparisons of locking strategies indicate heterogeneity in efficacy and safety across candidate solutions, underscoring that “one-size-fits-all” lock protocols may be suboptimal across access types and clinical contexts (Jalili et al., 2025). Central venous catheter dysfunction remains a common challenge in maintenance hemodialysis and may compromise prescribed blood flow, dialysis adequacy, and catheter survival (Lee et al., 2012; Yaxley et al., 2020). Urokinase-based locking or thrombolytic strategies are frequently used for intraluminal catheter dysfunction; however, clinical outcomes may also be influenced by the lock-solution diluent and by procedural factors such as dwell time, locking volume, and aspiration technique (Bonkain et al., 2021; Wang and Sun, 2022). At present, limited real-world evidence is available on whether using sodium bicarbonate rather than saline as the urokinase diluent is associated with improved catheter patency and safety outcomes when the dwell-and-aspiration procedure is standardized (Bau et al., 2023; Wathanavasin et al., 2021; El-Hennawy et al., 2019).

To address this evidence gap, we retrospectively evaluated catheter dysfunction, dialysis performance, procedural response, and safety outcomes in maintenance hemodialysis patients receiving a sodium bicarbonate–urokinase lock compared with a saline–urokinase lock under a standardized institutional protocol.

## 2 Methods

### 2.1 Study design and participants

This retrospective, single-center cohort study enrolled 220 adult patients (age >18 years) with an indwelling hemodialysis central venous catheter who underwent maintenance hemodialysis at our institution between December 2023 and March 2024. All included patients were receiving regular hemodialysis and were clinically stable during the study period. According to the predefined catheter locking schedule implemented in routine practice, patients were assigned to an observation group (sodium bicarbonate combined with urokinase) or a control group (0.9% sodium chloride combined with urokinase). Patients were excluded if they had evidence of catheter-related infection (exit-site infection, tunnel infection, or catheter-related bloodstream infection), active bleeding or a clinically relevant bleeding tendency/severe coagulopathy, known hypersensitivity to urokinase or sodium bicarbonate, catheter malposition or mechanical dysfunction requiring immediate replacement, confirmed catheter-associated thrombosis or central venous stenosis/occlusion requiring endovascular management, pregnancy, or incomplete records precluding endpoint assessment. Informed consent was obtained from all participants. The study was reviewed and approved by the hospital’s ethics committee and conducted in accordance with institutional guidelines and the Declaration of Helsinki. All data were anonymized prior to analysis to ensure participant confidentiality.

### 2.2 Catheter locking protocols and negative-pressure aspiration procedure

All patients used the same institutional hemodialysis CVC specification, including the same catheter brand, model, and clinical type. Catheter insertion, routine maintenance, locking volume determination, and negative-pressure aspiration were performed according to standardized institutional protocols. Thus, the primary procedural difference between groups was the diluent used for urokinase preparation: 5% sodium bicarbonate in the observation group and 0.9% sodium chloride in the control group. For the observation protocol, 100,000 units of urokinase were dissolved in 10 mL of 5% sodium bicarbonate solution to prepare a locking solution with a final concentration of 10,000 U/mL; for the control protocol, 100,000 units of urokinase were dissolved in 10 mL of 0.9% sodium chloride to achieve the same final concentration. Using strict aseptic technique, the prepared locking solution was instilled into each catheter lumen at 1.1–1.2 times the manufacturer-specified lumen fill (priming) volume and allowed to dwell for 30 min. After the dwell, negative-pressure aspiration was performed using an empty syringe by retracting the plunger to 2–3 mL to generate an estimated negative pressure of approximately 200–300 mmHg; the lock solution was aspirated and discarded, followed by aspiration of an additional 2–3 mL of blood to inspect for thrombus fragments and/or fibrin sheath material. When white fibrin sheath material was observed during aspiration, the process was documented by real-time video recording to capture dissolution and removal (Wang and Sun, 2022; Islam, 2024). At the end of the dialysis session, the same regimen was applied using the same preparation, instillation volume, and a 30-min dwell; the control group underwent an identical procedure, differing only in the diluent used for urokinase preparation.

### 2.3 Data collection

Data were extracted retrospectively from the electronic medical record system, hemodialysis treatment documentation, and catheter management/nursing records for all eligible patients during the study period (December 2023 to March 2024). A standardized case report form was used for data abstraction, and two investigators independently verified key variables related to exposure classification and outcomes; discrepancies were resolved by consensus review of the source records. Collected variables included: (1) demographic and baseline clinical characteristics (age, sex, body mass index, primary cause of end-stage kidney disease when available, hemodialysis vintage, and dialysis frequency); (2) comorbidities documented at enrollment (hypertension, diabetes mellitus, coronary artery disease and/or heart failure, atrial fibrillation, peripheral vascular disease, and other clinically relevant conditions recorded in the medical history); (3) laboratory indices obtained closest to enrollment or the index catheter intervention (platelet count, international normalized ratio, activated partial thromboplastin time, D-dimer, and fibrinogen); and (4) catheter-related characteristics (catheter type [tunneled vs. non-tunneled], lumen configuration, insertion site, catheter dwell time from insertion to study entry, and documented history of catheter dysfunction and prior thrombolysis/locking procedures).

Exposure data comprised the catheter locking regimen prescribed and administered according to the institutional schedule, including the diluent used for urokinase preparation (5% sodium bicarbonate vs. 0.9% sodium chloride), urokinase dose and concentration (100,000 U in 10 mL; 10,000 U/mL), the instilled volume (1.1–1.2 times the manufacturer-specified lumen fill volume), dwell duration (30 min), and the use of negative-pressure aspiration. Procedure-related observations were recorded from standardized nursing notes, including whether visible blood clots and/or fibrin sheath material were present in aspirated contents and whether dissolution of white fibrin sheath material was captured by real-time video documentation.

Outcome data were collected longitudinally across the observation window from dialysis session records and catheter management notes, including catheter dysfunction episodes, total number of dysfunction events, blood flow failure to achieve the prescribed extracorporeal blood flow, arterial/venous pressure abnormalities and pressure-alarm episodes, line reversal events, dialysis adequacy indices (Kt/V and urea reduction ratio), need for repeat thrombolytic procedures, and requirement for catheter exchange or reinsertion. Safety outcomes were abstracted from medical and nursing records and included bleeding-related adverse events, infection-related outcomes, antibiotic use for suspected or confirmed catheter infection, catheter removal or exchange due to infection, other adverse reactions, and dialysis interruption or delay attributable to adverse events. In addition, laboratory safety indicators were collected from routine predialysis blood tests during the observation period, including serum sodium, serum bicarbonate or total carbon dioxide, serum potassium, and serum chloride. Hypernatremia was defined as serum sodium >145 mmol/L, and elevated bicarbonate/total CO_2_ was defined as a value above the upper limit of the local laboratory reference range; for the present analysis, a threshold of >29 mmol/L was used.

### 2.4 Definition of catheter dysfunction and outcome assessment

Catheter dysfunction was defined according to predefined objective criteria derived from dialysis-session records and the institutional catheter-management protocol. A dysfunction episode was identified when the catheter failed to achieve or maintain an extracorporeal blood flow rate of ≥300 mL/min, when the prescribed blood flow could not be maintained because of catheter-related flow limitation, or when recurrent arterial/venous pressure alarms or pressure abnormalities required intervention. Additional criteria included the need for arterial–venous line reversal, inadequate aspiration or flushing of the catheter lumen, or dialysis interruption/delay attributable to insufficient catheter flow. Events caused by catheter malposition, kinking, fracture, confirmed central venous stenosis/occlusion, or catheter-related infection were not classified as functional catheter dysfunction, as these conditions were predefined exclusion criteria.

All catheter assessments and interventions were performed in the same hemodialysis department by trained nurses and physicians following the same institutional protocol. Catheter performance, achieved blood flow, pressure alarms, line reversal, thrombolytic intervention, and catheter exchange/reinsertion were documented using standardized dialysis and nursing records. During retrospective data abstraction, two investigators independently verified exposure and outcome variables, and discrepancies were resolved by consensus to minimize operator- and documentation-related variability.

### 2.5 Statistical analysis

All analyses were performed using IBM SPSS Statistics, version 26.0 (IBM Corp., Armonk, NY, USA). Continuous variables were assessed for distributional characteristics and are presented as mean ± standard deviation or median (interquartile range), as appropriate. Between-group comparisons were performed using the independent-samples t-test or Mann–Whitney U test. Categorical variables are expressed as counts and percentages and were compared using the Pearson chi-square test or Fisher’s exact test. The main outcome of interest was the occurrence of at least one catheter dysfunction episode during the observation period. Event-based outcomes, including total catheter dysfunction events and pressure-alarm episodes, were expressed as incidence rates per 1,000 catheter-days and per 100 hemodialysis sessions; between-group rate comparisons were performed using a Poisson rate approach, with incidence rate ratios and 95% confidence intervals reported. Procedural effectiveness and safety outcomes were analyzed using chi-square or Fisher’s exact tests, and dialysis adequacy indices were compared as continuous variables. Subgroup analyses stratified by insertion site, catheter dwell time, and catheter type were considered exploratory. Multivariable logistic regression was used to adjust for clinically relevant covariates and reduce potential confounding. Covariates were selected *a priori* based on clinical relevance and potential confounding effects, rather than by automated forward, backward, or stepwise selection, and were entered simultaneously into the model. The adequacy of the sample size for the primary multivariable logistic regression model was assessed using the events-per-variable (EPV) approach. All tests were two-sided, and P < 0.05 was considered statistically significant.

## 3 Results

### 3.1 Patient screening, exclusion, and grouping

**FIGURE 1:**
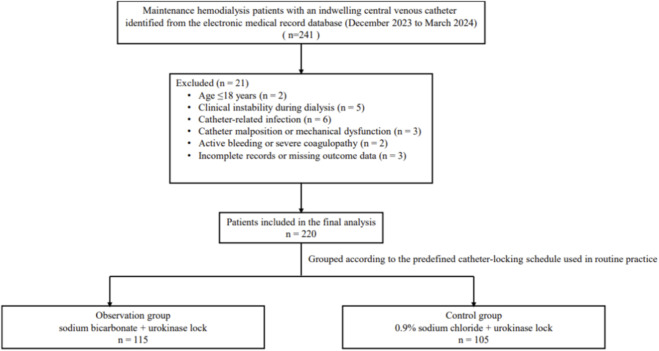
Flow diagram of patient screening, exclusion, and group allocation.

During the study period from December 2023 to March 2024, 241 maintenance hemodialysis patients with an indwelling central venous catheter were retrospectively identified from the electronic medical record database and screened for eligibility. After exclusion of 21 patients for predefined reasons, 220 eligible patients were included in the final analysis (Figure 1). Patients were grouped according to the predefined catheter-locking schedule used in routine clinical practice: 115 patients received sodium bicarbonate plus urokinase lock, and 105 patients received 0.9% sodium chloride plus urokinase lock. Catheter performance and subsequent management were assessed using standardized dialysis treatment records and catheter management documentation throughout the observation period.

### 3.2 Baseline demographic, clinical, and catheter-related characteristics

The two groups were comparable with respect to demographic variables, dialysis-related parameters, comorbidity burden, coagulation indices, and catheter-related characteristics (all P > 0.05; Table 1). The mean age was 62.65 ± 8.64 years in the observation group and 63.73 ± 9.42 years in the control group (t = −0.88, P = 0.380), with similar proportions of male patients (53.9% vs. 53.3%; χ^2^ = 0.01, P = 0.931) and comparable BMI (23.51 ± 3.24 vs. 23.15 ± 2.74 kg/m^2^; t = 0.91, P = 0.366). Hemodialysis vintage and the distribution of dialysis frequency were not significantly different between groups (both P > 0.05). The prevalence of hypertension, diabetes mellitus, coronary artery disease/heart failure, atrial fibrillation, and peripheral vascular disease showed no statistically significant differences. Baseline coagulation-related parameters (platelet count, INR, APTT, D-dimer, and fibrinogen) were similar between groups (all P > 0.05). Catheter characteristics, including the proportion of tunneled (semi-permanent) catheters, lumen configuration, and catheter dwell time before enrollment, were also comparable between groups. The median catheter dwell time before enrollment was 52.20 days [34.17, 73.24] in the observation group and 49.03 days [33.82, 77.49] in the control group, with no statistically significant difference (Z = 0.12, P = 0.905). Histories of catheter dysfunction and prior thrombolysis/locking procedures were also similar between groups (all P > 0.05) (Table 1).

**TABLE 1:** Baseline demographic and clinical characteristics.

Variable	Observation (n = 115)	Control (n = 105)	Test statistic	P Value
Age, years	62.65 ± 8.64	63.73 ± 9.42	t = −0.88	0.380
Male sex, n (%)	62 (53.9)	56 (53.3)	χ^2^ = 0.01	0.931
BMI, kg/m^2^	23.51 ± 3.24	23.15 ± 2.74	t = 0.91	0.366
Hemodialysis vintage, years	4.67 [3.63, 6.66]	5.23 [3.57, 7.27]	Z = −1.15	0.250
Dialysis frequency, 3 sessions/week, n (%)	103 (89.6)	96 (91.4)	χ^2^ = 0.22	0.638
Hypertension, n (%)	68 (59.1)	59 (56.2)	χ^2^ = 0.19	0.659
Diabetes mellitus, n (%)	33 (28.7)	32 (30.5)	χ^2^ = 0.08	0.772
Coronary artery disease/heart failure, n (%)	18 (15.7)	23 (21.9)	χ^2^ = 1.42	0.234
Atrial fibrillation, n (%)	12 (10.4)	11 (10.5)	χ^2^ = 0.00	0.992
Peripheral vascular disease, n (%)	16 (13.9)	9 (8.6)	χ^2^ = 1.55	0.212
Platelet count, ×10^9^/L	225.45 ± 56.38	217.21 ± 55.39	t = 1.09	0.276
INR	1.06 ± 0.12	1.05 ± 0.13	t = 0.43	0.670
APTT, s	32.40 ± 4.38	33.37 ± 4.42	t = −1.63	0.104
D-dimer, mg/L FEU	0.88 [0.58, 1.29]	1.07 [0.59, 1.47]	Z = −1.27	0.204
Fibrinogen, g/L	3.41 ± 0.60	3.35 ± 0.75	t = 0.72	0.470
Tunneled (semi-permanent) catheter, n (%)	91 (79.1)	82 (78.1)	χ^2^ = 0.04	0.852
Double-lumen catheter, n (%)	110 (95.7)	101 (96.2)	Fisher	1.000
Catheter dwell time before enrollment, days	52.20 [34.17, 73.24]	49.03 [33.82, 77.49]	Z = 0.12	0.905
History of catheter dysfunction, n (%)	27 (23.5)	31 (29.5)	χ^2^ = 1.03	0.309
History of thrombolysis/locking, n (%)	31 (27.0)	19 (18.1)	χ^2^ = 2.45	0.117
Catheter dwell time before enrollment, days	52.20 [34.17, 73.24]	49.03 [33.82, 77.49]	Z = 0.12	0.905

APTT, activated partial thromboplastin time; BMI, body mass index; FEU, fibrinogen-equivalent units; INR, international normalized ratio.

### 3.3 Catheter dysfunction incidence and dialysis performance

During the observation window, the proportion of patients experiencing at least one catheter dysfunction episode was lower in the observation group than in the control group (20.9% vs. 34.3%; χ^2^ = 4.98, P = 0.026; Table 2). The observation group also had fewer total catheter dysfunction events (35 vs. 61), corresponding to lower incidence rates per 1,000 catheter-days (4.35 vs. 8.30) and per 100 hemodialysis sessions (0.64 vs. 1.22), with an incidence rate ratio of 0.52 (Z = −3.05, P = 0.002). For catheter performance indicators, prescribed blood flow failure was observed in 16.5% of patients in the observation group and 27.6% in the control group (χ^2^ = 3.96, P = 0.047). Arterial/venous pressure abnormalities occurred in 24.3% and 39.0% of patients, respectively (χ^2^ = 5.51, P = 0.019), and pressure-abnormality episodes were less frequent in the observation group when expressed per 100 sessions (1.68 vs. 2.91; IRR = 0.58, Z = −4.08, P < 0.001). Line reversal was required in 12.2% of patients in the observation group and 22.9% in the control group (χ^2^ = 4.38, P = 0.036). Dialysis adequacy indices were higher in the observation group, including Kt/V (1.46 ± 0.20 vs. 1.39 ± 0.22; t = 2.46, P = 0.015) and URR (70.2% ± 6.0% vs. 67.9% ± 6.5%; t = 2.72, P = 0.007) (Table 2).

**TABLE 2:** Catheter dysfunction incidence and dialysis performance outcomes.

Outcome	Observation (n = 115)	Control (n = 105)	Test statistic	P Value
Total catheter-days, days	8,050	7,350	—	—
Total hemodialysis sessions, sessions	5,460	4,980	—	—
Patients with ≥1 catheter dysfunction episode, n (%)	24 (20.9)	36 (34.3)	χ^2^ = 4.98	0.026
Catheter dysfunction events, total (rate per 1,000 catheter-days; per 100 sessions)	35 (4.35; 0.64)	61 (8.30; 1.22)	Z = −3.05 (IRR = 0.52)	0.002
Prescribed blood flow failure (≥1 episode), n (%)	19 (16.5)	29 (27.6)	χ^2^ = 3.96	0.047
Arterial/venous pressure abnormality (≥1 episode), n (%)	28 (24.3)	41 (39.0)	χ^2^ = 5.51	0.019
Arterial/venous pressure abnormality episodes, total (rate per 100 sessions)	92 (1.68)	145 (2.91)	Z = −4.08 (IRR = 0.58)	<0.001
Line reversal required (≥1 episode), n (%)	14 (12.2)	24 (22.9)	χ^2^ = 4.38	0.036
Kt/V	1.46 ± 0.20	1.39 ± 0.22	t = 2.46	0.015
URR, %	70.2 ± 6.0	67.9 ± 6.5	t = 2.72	0.007

IRR, incidence rate ratio; Kt/V, dialysis dose index (clearance × time/volume of distribution); URR, urea reduction ratio.

### 3.4 Intervention efficacy: restoration of catheter patency and thrombolytic response

A total of 96 thrombolytic locking/aspiration procedures were recorded for suspected catheter dysfunction during the observation period, including 35 episodes in the observation group and 61 episodes in the control group. After the 30-min dwell, immediate restoration of catheter patency with achievement of the prescribed extracorporeal blood flow was observed more frequently in the observation group than in the control group (85.7% vs. 65.6%; χ^2^ = 4.57, P = 0.033; Table 3). Repeat intervention, defined as an additional thrombolytic procedure during the same dialysis session or a subsequent session because of incomplete restoration, was recorded in 11.4% of episodes in the observation group and 32.8% in the control group (χ^2^ = 5.41, P = 0.020). Visible blood clots and/or fibrin sheath material on negative-pressure aspiration were documented less frequently in the observation group than in the control group (22.9% vs. 45.9%; χ^2^ = 5.04, P = 0.025). Among episodes with visible fibrin sheath material, video-documented white fibrin sheath dissolution during aspiration was observed in 5 of 6 episodes (83.3%) in the observation group and 4 of 18 episodes (22.2%) in the control group (Fisher’s exact test, P = 0.015). In the pooled analysis of 60 patients who developed catheter dysfunction, visible clot and/or fibrin sheath material during aspiration was associated with a higher proportion of subsequent recurrent catheter dysfunction after initial restoration (58.3% vs. 16.7%; OR = 7.00, 95% CI 2.12–23.11; χ^2^ = 11.25, P = 0.001; Table 4). Catheter exchange or reinsertion was required in 2 patients (1.7%) in the observation group and 9 patients (8.6%) in the control group (χ^2^ = 5.39, P = 0.020).

**TABLE 3:** Procedural effectiveness and aspiration findings during thrombolytic interventions.

Outcome (per dysfunction episode unless otherwise specified)	Observation group	Control group	Test statistic	P Value
Thrombolytic locking/aspiration procedures, n (episodes)	35	61	—	—
Single-procedure success after 30-min dwell, n (%)	30 (85.7)	40 (65.6)	χ^2^ = 4.57	0.033
Repeat intervention required, n (%)	4 (11.4)	20 (32.8)	χ^2^ = 5.41	0.020
Visible blood clot and/or fibrin sheath material on aspiration, n (%)	8 (22.9)	28 (45.9)	χ^2^ = 5.04	0.025
Visible fibrin sheath material on aspiration, n (%)	6 (17.1)	18 (29.5)	χ^2^ = 1.81	0.178
Video-documented white fibrin sheath dissolution (among fibrin-sheath–positive episodes), n (%)	5/6 (83.3)	4/18 (22.2)	Fisher	0.015
Catheter exchange/reinsertion required (per patient), n (%)	2/115 (1.7)	9/105 (8.6)	χ^2^ = 5.39	0.020

CVC, central venous catheter.

**TABLE 4:** Association between aspiration findings and recurrent catheter dysfunction.

Variable	Recurrent dysfunction, n/N (%)	No recurrence, n/N (%)	Effect estimate	Test statistic	P Value
Visible clot/fibrin sheath present (n = 24 patients)	14/24 (58.3)	10/24 (41.7)	OR = 7.00 (95% CI 2.12–23.11)	χ^2^ = 11.25	0.001
Visible clot/fibrin sheath absent (n = 36 patients)	6/36 (16.7)	30/36 (83.3)	Reference	—	—

OR, odds ratio; CI, confidence interval.

### 3.5 Laboratory safety indicators, complications, and safety outcomes

Laboratory safety indicators were comparable between groups (Table 5). Serum sodium, serum bicarbonate/total CO_2_, potassium, and chloride levels showed no significant between-group differences, and the incidences of hypernatremia and elevated bicarbonate/total CO_2_ were also similar (all P > 0.05). Bleeding-related adverse events did not differ significantly between groups. Any bleeding event occurred in 14 patients (12.2%) in the observation group and 19 patients (18.1%) in the control group (P = 0.219). Access-site oozing, subcutaneous hematoma, gastrointestinal bleeding, intracranial hemorrhage, and major bleeding were uncommon and comparable between groups. Infection-related events were infrequent. Catheter-related bloodstream infection occurred in 1 patient (0.9%) in the observation group and 7 patients (6.7%) in the control group (P = 0.029), while exit-site infection occurred in 3 patients (2.6%) and 10 patients (9.5%), respectively (P = 0.043). Antibiotic use for suspected or confirmed catheter infection was recorded in 8 patients (7.0%) and 17 patients (16.2%), respectively (P = 0.031). Given the small number of events, these infection-related findings were interpreted descriptively. Other adverse reactions, including hypersensitivity, chest discomfort, and dialysis interruption or delay attributable to adverse events, were rare and showed no significant between-group differences.

**TABLE 5:** Laboratory safety indicators, complications, and safety outcomes.

Outcome	Observation (n = 115)	Control (n = 105)	Test statistic	P Value
Laboratory safety indicators				
Serum sodium, mmol/L	138.6 ± 3.2	138.2 ± 3.5	t = 0.88	0.379
Serum bicarbonate/total CO_2_, mmol/L	23.8 ± 2.9	23.5 ± 3.0	t = 0.75	0.452
Serum potassium, mmol/L	4.72 ± 0.55	4.78 ± 0.60	t = −0.77	0.442
Serum chloride, mmol/L	100.8 ± 3.6	101.1 ± 3.8	t = −0.60	0.549
Hypernatremia, n (%)	3 (2.6)	2 (1.9)	Fisher	1.000
Elevated bicarbonate/total CO_2_, n (%)	4 (3.5)	2 (1.9)	Fisher	0.685
Bleeding-related adverse events				
Any bleeding event, n (%)	14 (12.2)	19 (18.1)	χ^2^ = 1.51	0.219
Access-site oozing, n (%)	9 (7.8)	11 (10.5)	χ^2^ = 0.47	0.495
Subcutaneous hematoma, n (%)	4 (3.5)	5 (4.8)	Fisher	0.740
Gastrointestinal bleeding, n (%)	1 (0.9)	2 (1.9)	Fisher	0.607
Intracranial hemorrhage, n (%)	0 (0.0)	1 (1.0)	Fisher	0.477
Major bleeding, n (%)	1 (0.9)	3 (2.9)	Fisher	0.350
Infection-related outcomes				
CRBSI, n (%)	1 (0.9)	7 (6.7)	Fisher	0.029
Exit-site infection, n (%)	3 (2.6)	10 (9.5)	Fisher	0.043
Tunnel infection, n (%)	1 (0.9)	4 (3.8)	Fisher	0.195
Antibiotic use for suspected/confirmed catheter infection, n (%)	8 (7.0)	17 (16.2)	χ^2^ = 4.65	0.031
Catheter removal/exchange due to infection, n (%)	1 (0.9)	4 (3.8)	Fisher	0.195
Other adverse reactions and tolerability				
Hypersensitivity reaction, n (%)	1 (0.9)	1 (1.0)	Fisher	1.000
Chest discomfort during/after procedure, n (%)	3 (2.6)	4 (3.8)	Fisher	0.712
Dialysis interruption/delay attributable to adverse events, n (%)	4 (3.5)	9 (8.6)	Fisher	0.153

Abbreviations: CRBSI, catheter-related bloodstream infection; GI, gastrointestinal; total CO_2_, total carbon dioxide.

### 3.6 Exploratory subgroup analyses stratified by catheter characteristics

Exploratory subgroup analyses were performed according to insertion site, pre-enrollment catheter dwell time, and catheter type (Table 6). For the main outcome of at least one catheter dysfunction episode, the observed proportions were numerically lower in the observation group across the internal jugular vein subgroup (17.5% vs. 28.6%; RR = 0.61; P = 0.106), femoral vein subgroup (28.6% vs. 45.7%; RR = 0.62; P = 0.138), and both dwell-time categories (<60 days: 17.1% vs. 30.6%; P = 0.068; ≥60 days: 26.7% vs. 39.5%; P = 0.199). Among patients with tunneled catheters, the between-group difference reached nominal statistical significance (18.7% vs. 31.7%; RR = 0.59; P = 0.048), whereas no statistically significant difference was observed in the non-tunneled catheter subgroup (29.2% vs. 43.5%; P = 0.307). For episode-level procedural outcomes, single-procedure success after the standardized 30-min dwell was numerically higher in the observation group across subgroups. The difference reached nominal statistical significance in the internal jugular vein subgroup (90.9% vs. 66.7%; RR = 1.36; P = 0.036), while the remaining subgroup comparisons did not reach statistical significance. Given the limited number of patients and events within each stratum, these subgroup findings were considered exploratory and interpreted descriptively.

**TABLE 6:** Exploratory subgroup analyses of catheter dysfunction and episode-level procedural outcomes by catheter characteristics.

Stratification factor	Subgroup	Outcome	Observation, n/N (%)	Control, n/N (%)	Effect estimate	Test statistic	P Value
Insertion site	Internal jugular vein	Patients with ≥1 dysfunction episode	14/80 (17.5)	20/70 (28.6)	RR = 0.61 (95% CI 0.34–1.12)	χ^2^ = 2.61	0.106
	Internal jugular vein	Single-procedure success after 30-min dwell	20/22 (90.9)	24/36 (66.7)	RR = 1.36 (95% CI 1.05–1.78)	χ^2^ = 4.38	0.036
	Femoral vein	Patients with ≥1 dysfunction episode	10/35 (28.6)	16/35 (45.7)	RR = 0.62 (95% CI 0.33–1.18)	χ^2^ = 2.20	0.138
	Femoral vein	Single-procedure success after 30-min dwell	10/13 (76.9)	16/25 (64.0)	RR = 1.20 (95% CI 0.79–1.83)	Fisher	0.486
Catheter dwell time (pre-enrollment)	<60 days	Patients with ≥1 dysfunction episode	12/70 (17.1)	19/62 (30.6)	RR = 0.56 (95% CI 0.30–1.06)	χ^2^ = 3.34	0.068
	<60 days	Single-procedure success after 30-min dwell	18/20 (90.0)	24/34 (70.6)	RR = 1.27 (95% CI 0.98–1.66)	Fisher	0.174
	≥60 days	Patients with ≥1 dysfunction episode	12/45 (26.7)	17/43 (39.5)	RR = 0.67 (95% CI 0.37–1.24)	χ^2^ = 1.65	0.199
	≥60 days	Single-procedure success after 30-min dwell	12/15 (80.0)	16/27 (59.3)	RR = 1.35 (95% CI 0.90–2.02)	χ^2^ = 1.87	0.172
Catheter type	Tunneled	Patients with ≥1 dysfunction episode	17/91 (18.7)	26/82 (31.7)	RR = 0.59 (95% CI 0.35–1.00)	χ^2^ = 3.92	0.048
	Tunneled	Single-procedure success after 30-min dwell	23/26 (88.5)	30/44 (68.2)	RR = 1.30 (95% CI 1.02–1.66)	χ^2^ = 3.66	0.056
	Non-tunneled	Patients with ≥1 dysfunction episode	7/24 (29.2)	10/23 (43.5)	RR = 0.67 (95% CI 0.31–1.46)	χ^2^ = 1.04	0.307
	Non-tunneled	Single-procedure success after 30-min dwell	7/9 (77.8)	10/17 (58.8)	RR = 1.32 (95% CI 0.78–2.24)	Fisher	0.418

CI, confidence interval; RR, risk ratio.

### 3.7 Multivariable analyses of catheter dysfunction occurrence and recurrence

In Model 1, multivariable logistic regression was used to evaluate factors associated with the occurrence of catheter dysfunction in the full cohort (dependent variable: ≥1 dysfunction episode during the observation window; n = 220). After adjustment for clinically relevant covariates, the sodium bicarbonate–urokinase locking regimen was associated with lower odds of catheter dysfunction compared with the 0.9% sodium chloride–urokinase regimen (adjusted odds ratio [aOR] = 0.49, 95% CI 0.26–0.90; P = 0.024). Femoral access was associated with higher odds of dysfunction than internal jugular access (aOR = 1.95, 95% CI 1.02–3.75; P = 0.044), and higher baseline D-dimer levels were also associated with higher odds of dysfunction (per 1 mg/L FEU increase: aOR = 1.28, 95% CI 1.04–1.58; P = 0.021). Catheter dwell time ≥60 days, hemodialysis vintage, and diabetes mellitus were not statistically significant in the adjusted model. In Model 2, an exploratory multivariable logistic regression was performed among patients who experienced dysfunction and achieved initial restoration (n = 60) to evaluate factors associated with recurrent dysfunction. Visible blood clot and/or fibrin sheath material during negative-pressure aspiration was associated with higher odds of recurrence (aOR = 5.62, 95% CI 1.91–16.55; P = 0.002). The locking regimen was not statistically significant in this recurrence model (P = 0.145) (Table 7). For Model 1, the primary multivariable adjusted analysis of catheter dysfunction occurrence included 60 events and six clinically prespecified covariates, yielding an EPV of 10.0. This suggested that the sample size was acceptable for confounding-adjusted logistic regression. For Model 2, the recurrence analysis included 20 recurrent events and five covariates, yielding an EPV of 4.0. Therefore, Model 2 was considered exploratory, and its results should be interpreted cautiously.

**TABLE 7:** Multivariable logistic regression for catheter dysfunction occurrence and recurrence.

Predictor	aOR (95% CI)	P Value
Model 1: Occurrence of catheter dysfunction (n = 220)	​	​
Locking regimen (sodium bicarbonate + urokinase vs. 0.9% sodium chloride + urokinase)	0.49 (0.26–0.90)	0.024
Insertion site (femoral vs. internal jugular)	1.95 (1.02–3.75)	0.044
Pre-enrollment catheter dwell time (≥60 days vs. < 60 days)	1.62 (0.88–2.99)	0.122
Hemodialysis vintage (per 1-year increase)	1.08 (0.98–1.19)	0.120
Diabetes mellitus (yes vs. no)	1.41 (0.78–2.57)	0.259
D-dimer (per 1 mg/L FEU increase)	1.28 (1.04–1.58)	0.021
Model 2: Recurrence after initial restoration among dysfunction patients (n = 60)	​	​
Visible clot and/or fibrin sheath material on aspiration (yes vs. no)	5.62 (1.91–16.55)	0.002
Locking regimen (sodium bicarbonate + urokinase vs. 0.9% sodium chloride + urokinase)	0.46 (0.16–1.29)	0.145
Insertion site (femoral vs. internal jugular)	1.72 (0.62–4.77)	0.297
Pre-enrollment catheter dwell time (≥60 days vs. < 60 days)	1.55 (0.57–4.23)	0.391
Diabetes mellitus (yes vs. no)	1.49 (0.52–4.25)	0.457

aOR, adjusted odds ratio; CI, confidence interval; FEU, fibrinogen-equivalent units.

## 4 Discussion

In this single-center retrospective cohort, the sodium bicarbonate–urokinase admixture was associated with lower observed rates of hemodialysis CVC dysfunction and higher dialysis adequacy indices compared with saline–urokinase locking. At the patient level, at least one dysfunction episode was recorded in 34.3% of patients in the saline–urokinase group and 20.9% in the sodium bicarbonate–urokinase group, with a similar direction of findings across several catheter performance indicators. At the event level, total dysfunction episodes and standardized incidence rates were also lower in the sodium bicarbonate–urokinase group, indicating fewer recorded dysfunction events during the observation window. In parallel, Kt/V and urea reduction ratio were modestly higher in this group. These findings suggest that improved catheter performance indicators may be clinically relevant, as catheter dysfunction is recognized as a common contributor to inadequate dialysis delivery and subsequent access-related interventions (Lok et al., 2025).

The adjusted and exploratory stratified analyses provide further context for interpreting the observed catheter-performance findings. In the multivariable model, the sodium bicarbonate–urokinase regimen remained associated with lower odds of catheter dysfunction after adjustment for clinically relevant covariates, whereas femoral access and higher baseline D-dimer were associated with higher odds of dysfunction (Wathanavasin et al., 2021; Van Hulle et al., 2019). This pattern is clinically plausible because catheter performance is influenced by both local access-related factors and the systemic coagulation milieu (Wang et al., 2024). Femoral catheters may be more susceptible to flow disturbance because of anatomical and positional factors, while elevated D-dimer may reflect a prothrombotic state that predisposes to intraluminal fibrin or thrombotic obstruction. Exploratory subgroup analyses showed a broadly similar direction of observed differences across insertion site, catheter dwell-time categories, and catheter type, although several comparisons did not reach statistical significance. These findings suggest that the association between the locking regimen and catheter performance was not confined to a single catheter subgroup. However, the subgroup results should be interpreted descriptively because the numbers of patients and events within individual strata were limited. Taken together, the adjusted and subgroup findings support the relevance of considering lock-solution composition together with access site, catheter duration, and coagulation status when evaluating real-world CVC management strategies (Tomaszek and Rahman, 2024).

The procedural findings add clinically relevant information beyond patient-level dysfunction incidence. Among thrombolytic locking/aspiration procedures performed for suspected dysfunction, immediate restoration of catheter patency after the 30-min dwell was observed more frequently in the sodium bicarbonate–urokinase group, whereas repeat intervention and catheter exchange/reinsertion were less frequent. These findings indicate that the observed between-group differences extended to downstream catheter-management outcomes after dysfunction was recognized. Negative-pressure aspiration findings may also have practical value: visible blood clot and/or fibrin sheath material was documented less frequently in the sodium bicarbonate–urokinase group, and its presence was associated with recurrent dysfunction after initial restoration (Aitken et al., 2025; Waters et al., 2023). This suggests that aspiration findings may serve as a bedside marker for closer surveillance or further evaluation for fibrin sheath or central venous pathology when clinically appropriate (Kasradze et al., 2025). Safety outcomes were broadly comparable, with no significant between-group differences in bleeding events or laboratory indicators related to sodium, bicarbonate/total carbon dioxide, potassium, chloride, hypernatremia, or elevated bicarbonate/total carbon dioxide (Del Risco Zevallos et al., 2025). Infection-related events, including catheter-related bloodstream infection, exit-site infection, and antibiotic use for suspected or confirmed catheter infection, were numerically less frequent in the sodium bicarbonate–urokinase group; however, these findings were based on small event numbers and were interpreted descriptively (Lok et al., 2024).

Within the last 3 years, evidence regarding sodium bicarbonate locks has expanded across catheter types and comparators. In non-tunneled acute hemodialysis catheters, Ali et al. (Ali et al., 2023) reported similar safety and efficacy of a sodium bicarbonate lock versus an antibiotic-plus-heparin lock, including comparable catheter loss from dysfunction, CRBSI, and adequacy of blood flow. The BicarbLock trial compared 8.4% sodium bicarbonate with heparin (5,000 IU/mL) in non-tunneled catheters and reported comparable prevention of lumen thrombosis and catheter-related complications, supporting its feasibility as a heparin alternative in selected settings (Islam, 2024). In tunneled cuffed catheters, Kasradze et al. (Kasradze et al., 2025)observed lower CRBSI incidence with 8.4% sodium bicarbonate than with heparin and infection-free catheter survival comparable to an antibiotic-containing citrate lock, suggesting sodium bicarbonate as a potential non-antibiotic option when infection prevention is prioritized Parallel developments in lock-solution research also indicate that dedicated antimicrobial components may be associated with substantial reductions in CRBSI, as illustrated by the LOCK IT-100 study summary comparing taurolidine/heparin with heparin alone (Agarwal et al., 2025). Contemporary scoping work further emphasizes that lock selection is context dependent and may be influenced by infection risk, thrombosis risk, antimicrobial resistance concerns, and feasibility constraints (Alfieri et al., 2025). Against this background, the present study adds real-world evidence on a sodium bicarbonate–urokinase admixture used within a standardized dwell-and-aspiration protocol. The findings describe lower observed dysfunction burden, modestly higher dialysis adequacy indices, fewer documented visible clot/fibrin sheath findings, and less frequent escalation to catheter exchange in the sodium bicarbonate–urokinase group; however, these observations should be interpreted as associative and require prospective validation.

When pharmacologic intraluminal management is indicated for prevention or early management of CVC dysfunction, the sodium bicarbonate–urokinase regimen within a standardized 30-min dwell-and-aspiration protocol was associated with lower dysfunction incidence, improved achievement of prescribed blood flow, and modestly better dialysis adequacy. Visible clot/fibrin sheath material during aspiration may help identify patients at higher risk of recurrence who require closer surveillance or further evaluation for fibrin sheath or central venous pathology. The protocolized procedure, comprehensive outcome assessment, and multivariable adjustment for clinically relevant covariates support the interpretability of the findings. Consistent trends across blood flow failure, pressure abnormalities, line reversal, and adequacy indices also support the internal coherence of the results. From an infection-control perspective, the lower infection-related event rates should be interpreted cautiously because of the small number of events. Catheter-related infection is closely linked to microbial adhesion, biofilm formation, and virulence-factor expression. Zhou et al. (Zhou et al., 2024)reported that a non-antibiotic bioactive compound attenuated *Serratia marcescens* biofilm formation and virulence-related phenotypes, supporting the broader concept that biofilm/virulence modulation may contribute to catheter infection-control strategies. Therefore, the infection-related findings in the present study should be regarded as hypothesis-generating and require mechanistic and prospective clinical validation.

This study has several limitations. First, the retrospective, single-center, non-randomized design may have introduced selection bias, residual confounding, and practice-pattern effects, despite baseline comparisons, standardized data abstraction, and multivariable adjustment for clinically relevant covariates. Future multicenter prospective randomized trials are needed to validate these associative findings under more rigorous conditions. Second, although catheter dysfunction was defined using objective dialysis-record criteria, pressure-alarm documentation and catheter-management practices may vary across centers, which may limit external generalizability. Standardized multicenter definitions and harmonized catheter-performance recording would improve comparability across studies. Third, subgroup analyses by insertion site, catheter dwell time, and catheter type were exploratory and limited by small numbers of patients and events within each stratum. Larger studies are required to determine whether the observed associations are consistent across catheter-related subgroups. Fourth, rare safety outcomes, including major bleeding, tunnel infection, and catheter removal for infection, occurred infrequently, limiting the precision of these estimates. Future studies with larger sample sizes and longer follow-up are needed to better characterize uncommon safety events. Fifth, only the 5% sodium bicarbonate formulation used in routine institutional practice was evaluated; therefore, the optimal concentration remains undetermined. Future concentration-gradient studies, including 1.25%, 5%, and 8.4% sodium bicarbonate, may help clarify dose-response relationships. Sixth, mechanistic interpretation was limited because clot/fibrin sheath findings were based on visual assessment during aspiration rather than objective confirmation. Future studies incorporating vascular ultrasound, central venography, and serial thrombotic markers may better characterize fibrin sheath formation and thrombotic obstruction. Finally, the intervention combined lock-solution composition with a standardized dwell-and-aspiration procedure, and no heparin-based comparator was included. Future comparator or factorial studies including heparin-based and other standard lock solutions are warranted to clarify the relative contribution and clinical value of sodium bicarbonate–urokinase locking.

## 5 Conclusion

This retrospective single-center study suggests that, compared with saline–urokinase locking, sodium bicarbonate combined with urokinase within a standardized dwell-and-aspiration protocol may be associated with lower rates of hemodialysis CVC dysfunction, fewer dysfunction-related events, higher single-procedure patency restoration after a 30-min dwell, and less frequent repeat intervention or catheter exchange, with modestly higher dialysis adequacy indices. The safety profile appeared comparable between groups, with no significant increase in bleeding. The lower infection-related event rates should be interpreted cautiously because of the small number of events and should be considered hypothesis-generating.

## Data Availability

The raw data supporting the conclusions of this article will be made available by the authors, without undue reservation.
